# Lipoprotein(a) in Familial Hypercholesterolemia

**DOI:** 10.1016/j.cjco.2023.09.018

**Published:** 2023-09-30

**Authors:** Erin O. Jacob, Adam D. McIntyre, Jian Wang, Robert A. Hegele

**Affiliations:** a Department of Biochemistry, Schulich School of Medicine and Dentistry, Western University, London, Ontario, Canada; b Robarts Research Institute, Schulich School of Medicine and Dentistry, Western University, London, Ontario, Canada; c Department of Medicine, Schulich School of Medicine and Dentistry, Western University, London, Ontario, Canada

## Abstract

**Background:**

Low density lipoprotein (LDL) and Lipoprotein (Lp)(a) are proatherogenic apolipoprotein (apo) B-containing members of the non–high-density lipoprotein (non-HDL) family of particles. Elevated plasma levels of LDL cholesterol (C), non-HDL-C, and apo B are defining features of heterozygous familial hypercholesterolemia (HeFH), but reports of elevated plasma Lp(a) concentration are inconsistent.

**Methods:**

We performed retrospective chart reviews of 256 genetically characterized patients with hypercholesterolemia and 272 control subjects from the Lipid Genetics Clinic at University Hospital in London, Ontario. We evaluated pairwise correlations between plasma levels of Lp(a) and those of LDL-C, non-HDL-C and apo B.

**Results:**

Mean Lp(a) levels were not different between individuals with hypercholesterolemia and control subjects. No correlations were found between Lp(a) and LDL-C or non–HDL-C levels in controls or patients with hypercholesterolemia; all *r* values < 0.079 and all *P* values > 0.193. Borderline weak correlations between Lp(a) and apo B were identified in patients *r* = 0.103; *P* = 0.112) and controls (*r* = 0.175; *P* = 0.005). Results were similar across genotypic subgroups.

**Conclusions:**

Lp(a) levels are independent of LDL-C and non–HDL-C; in particular Lp(a) levels are not increased in patients with hypercholesterolemia and molecularly proven HeFH. Apo B was only weakly associated with Lp(a). Elevated Lp(a) does not cause FH in our clinic patients. Genetic variants causing HeFH that raise LDL-C do not affect Lp(a), confirming that these lipoproteins are metabolically distinct. Lp(a) cannot be predicted from LDL-C and must be determined separately to evaluate its amplifying effect on atherosclerotic risk in patients with hypercholesterolemia.

## Graphical abstract

**Figure:**
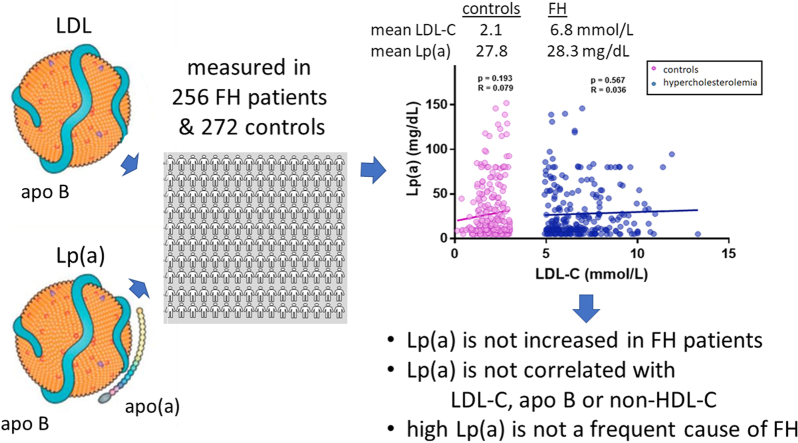

## Résumé

**Introduction:**

Les lipoprotéines de faible densité (LDL) et les lipoprotéines (Lp)(a) sont des éléments contenant des apolipoprotéines (apo)B proathérogènes de la famille des particules non à lipoprotéines de haute densité (non-HDL). Des concentrations plasmatiques élevées de cholestérol LDL, de cholestérol non-HDL et d’apoB sont des caractéristiques déterminantes de l’hypercholestérolémie familiale de type hétérozygote (HFHe), mais les documents qui portent sur les concentrations plasmatiques élevées de Lp(a) sont contradictoires.

**Méthodes:**

Nous avons effectué des revues rétrospectives de dossiers de 256 patients ayant les caractéristiques génétiques de l’hypercholestérolémie, et 272 témoins de la clinique de dyslipidémies génétiques de l’Hôpital universitaire de London, en Ontario. Nous avons évalué les corrélations par paires entre les concentrations plasmatiques de Lp(a) et celles du cholestérol LDL, du cholestérol non-HDL et de l’apoB.

**Résultats:**

Les concentrations moyennes de Lp(a) n’étaient pas différentes entre les individus atteints d’hypercholestérolémie et les témoins. Aucune corrélation n’a été observée entre les concentrations de Lp(a) et de cholestérol LDL ou de cholestérol non–HDL des témoins ou des patients atteints d’hypercholestérolémie ; toutes valeurs *r* < 0,079 et toutes valeurs *P* > 0,193. Nous avons relevé des corrélations limites faibles entre les concentrations de Lp(a) et d’apoB chez les patients *r* = 0,103 ; *P* = 0,112) et les témoins (*r* = 0,175 ; *P* = 0,005). Les résultats étaient similaires dans tous les sous-groupes génotypiques.

**Conclusions:**

Les concentrations de Lp(a) sont indépendantes des concentrations de cholestérol LDL et de cholestérol non–HDL ; notamment les concentrations de Lp(a) n’augmentent pas chez les patients atteints d’hypercholestérolémie et de HFHe moléculairement prouvée. L’apoB n’était que faiblement associée aux concentrations de Lp(a). Les concentrations élevées de Lp(a) ne causaient pas la HF chez les patients de notre clinique. Les variants génétiques causant la HFHe qui font augmenter les concentrations de cholestérol LDL n’affectent pas les concentrations de Lp(a), ce qui confirme que ces lipoprotéines sont distinctes sur le plan métabolique. Puisqu’on ne peut prédire les concentrations de Lp(a) à partir des concentrations de cholestérol LDL, elles doivent être déterminées séparément pour évaluer leur effet amplificateur sur le risque athérosclérotique des patients atteints d’hypercholestérolémie.

Heterozygous familial hypercholesterolemia (HeFH) is characterized by elevated plasma levels of the proatherogenic low-density lipoprotein (LDL). An estimated 1 in 250 to 300 people are affected with HeFH, making it among the most prevalent of genetic disorders.^1^ The inherited pathogenic variants typically result in dysfunctional LDL receptors (LDLRs) or interacting proteins, leading to improper clearance of LDL particles from the blood, and elevated LDL cholesterol (C) > 5.0 mmol/L.^1^ HeFH is clinically diagnosed using LDL-C levels, patient and family history, and physical findings, whereas genetic testing can confirm a definite diagnosis and identify causal variants.^1^ Monogenic HeFH is a condition in which a single deleterious variant compromises LDL catabolism. In contrast, in polygenic hypercholesterolemia, many small-effect variants (ie single nucleotide polymorphisms [SNPs]) affect synthesis or catabolism of LDL particles, raising LDL-C levels into the HeFH range.^2^, ^3^, ^4^ In addition, many patients with HeFH have neither a monogenic variant nor a high (> 90th percentile) polygenic risk score of identified small-effect variants. Irrespective of the underlying genetic cause, phenotypic HeFH with lifelong elevated LDL-C, if untreated, can lead to premature cardiovascular disease including heart attack, stroke, and aortic valve disease.^1^, ^2^, ^3^, ^4^

Lipoprotein (Lp)(a) is a distinctive plasma lipoprotein particle that is an independent risk factor for atherosclerotic cardiovascular disease (ASCVD).^5^ Because of the striking similarities in structure between Lp(a) and LDL, it is sometimes hypothesized that these 2 particles might share synthetic or catabolic pathways and that their plasma levels might be correlated, especially in patients with hypercholesterolemia. The cholesterol content and structure of the 2 particles are indeed very similar, characterized by an amphipathic shell surrounding a lipid core with a single molecule of apolipoprotein (apo) B attached. Apo B is a structural protein recognized by the LDLR allowing internalization of the particles into cells. In addition, Lp(a) contains a single molecule of apo(a) covalently linked to apo B. LDL metabolism is well studied, but that of Lp(a) remains largely unknown. Lp(a) is considered to be metabolically distinct from LDL,^5^ with accumulating evidence indicating Lp(a) is not cleared by the LDLR.

Elevated plasma levels of LDL-C, non–high-density lipoprotein (HDL)-C and apo B are defining features of HeFH, although reports of elevated plasma levels of Lp(a) are inconsistent. Lp(a) levels have sometimes been reported as being higher in FH compared with normolipidemic subjects, but this could reflect sampling bias rather than a true biochemical relationship.^6^ Evaluating Lp(a) in patient cohorts could help clarify how FH-associated variants, including genetically dysfunctional LDLRs, relate to Lp(a) levels. We evaluated whether Lp(a) levels are correlated with different components of the lipid profile—including LDL-C, apo B, and non–HDL-C—in clinic patients with hypercholesterolemia.

## Methods

### Study subjects

A retrospective chart review of patients from the Lipid Genetics Clinic at London Health Sciences Center, University Hospital (LHSC, UH) in London, Ontario, was performed. All patients provided informed consent and the project was approved by the Western University Research Ethics Board (project number 0379). The inclusion criteria consisted of available plasma lipid profiles, including LDL-C and Lp(a) values, with LDL-C values that were consistent with a clinical diagnosis of HeFH (ie, > 5.0 mmol/L). From this cohort, only those with all lipid profile components were included (ie, apo B and non-HDL-C). Specific methods used to select patients and identify cases of HeFH have been outlined previously.^2^ Our controls were a convenience sample comprising mainly healthy laboratory and clinic volunteers and unrelated spouses of study participants. Inclusion criteria for controls consisted of availability of relevant plasma lipid profile variables, LDL-C < 3 mmol/L, and plasma triglycerides < 2 mmol/L.

### Plasma lipid profiles

Total cholesterol, HDL-C, LDL-C, apo B and Lp(a) were determined from fasting blood samples, using routine protocols at the Core Biochemistry Laboratory at LHSC, UH, in London, Ontario. Lp(a) was determined using immunoprecipitation and turbidimetric detection with the Incstar Lp(a) test kit (Ciba-Corning, Mississauga, Ontario, Canada). Apo B was measured in a clinical laboratory, using a polyethylene glycol enhanced Advia immunoturbidometric assay (Siemens, Oakville, Ontario, Canada). Total cholesterol and HDL-C were determined via enzymatic colorimetric analysis and non–HDL-C was calculated by subtracting HDL-C from total cholesterol. LDL-C was calculated using the Friedewald formula.

Up to 3 LDL-C values were available for each patient: LDL-C on the day of the initial consult, imputed untreated LDL-C, and the highest known or patient-reported LDL-C recorded. Imputed LDL-C was calculated for patients on lipid-lowering medications, allowing for estimation of their baseline value off medication.^7^ A total of 69 patients were on medication (41 on rosuvastatin 20 or 40 mg daily, 22 on atorvastatin 40 or 80 mg daily, 7 on another statin, and 35 were also on ezetimibe 10 mg daily), thus requiring imputation of LDL-C, as described.^7^ For untreated patients, LDL-C on the day of the initial consult was used. If this value was markedly < 5.0 mmol/L, the highest known LDL-C value was used instead; with this approach, the lowest included value was 4.94 mmol/L. For patients already on medication, the imputed LDL-C value was used. An imputed LDL-C < 5 mmol/L was compared with the patient’s highest reported value, which, if ≥ 5 mmol/L, was used instead. The total cholesterol values used to calculate non–HDL-C were corrected based on the imputed LDL-C value included in the study.^7^ As there is no validated imputation process for apo B in patients with HeFH, the same scaling factor as for LDL-C was used to impute apo B.^7^ Lp(a), total cholesterol, HDL-C, and apo B levels were collected on the day of initial consult only. Lp(a) is stable throughout life and does not vary based on external factors such as diet or statin use, so no correction was necessary. No patient was taking an inhibitor of proprotein convertase subtilisin kexin type 9 (PCSK9) at assessment.

### Next-generation DNA sequencing

DNA from whole blood was extracted using established methods.^8^ Genotype of each patient— monogenic pathogenic variants and polygenic risk score (PRS)—was determined using the LipidSeq targeted sequencing panel run on the Illumina MiSeq DNA sequencer (Illumina Inc, San Diego, California, USA), using standard protocols of the London Regional Genomics Centre.^8^ LipidSeq targets gene loci to identify rare pathogenic variants causal for FH as well as the top 10 common SNPs to yield an individual’s PRS for LDL-C.^2^ A weighted PRS ≥ 1.96, or 90th percentile, was considered high.^2^ The cohort was next subdivided by genotype: FH variant-positive or variant-negative, and high or low PRS. As the presence of a rare variant is independent of PRS,^2^, ^3^, ^4^ each patient’s monogenic and polygenic status was determined separately, and the majority of patients were either variant-positive or variant-negative with a high PRS.

### Statistical analysis

Statistical analyses were performed using Microsoft Excel. Pairwise correlations between plasma levels of Lp(a) and LDL-C, apo B, and non–HDL-C were determined on the hypercholesterolemia and control cohorts. The hypercholesterolemia cohort was then subdivided based on genotype (ie, FH variant-positive, FH variant-negative and high PRS (≥ 1.96), and FH variant-negative and low PRS (< 1.96)). Pearson correlation coefficients and *P* values were determined in Excel, all Student's *t*-tests unpaired and 2-tailed. Mann-Whitney U tests were used when determining the *P* value for 2 medians of skewed variables such as Lp(a).

## Results

### Patient demographics and genotype

A total of 256 patients with a clinical diagnosis of HeFH and 272 normolipidemic controls had appropriate LDL-C, non–HDL-C, and Lp(a) values for analysis; 238 patients and 257 controls had available apo B levels. Clinical and demographic characteristics are described in Table 1, subdivided by sex in Supplemental Table S1. A total of 154 patients tested positive for a known FH pathogenic variant, whereas 101 were considered variant-negative. Two hundred fifty-one patients had their PRS determined, with 64 patients at or above the 90th percentile (PRS ≥ 1.96) and 187 patients below this threshold.

**Table 1:** Clinical and demographic characteristics study subjects

	Hypercholesterolemia patients (n = 256)	Controls (n = 272)	*P* value
Age (years)	43.8 ± 14.6	50.9 ± 14.9	4.25 × 10^-8^
BMI∗ (kg/m^2^)	26.8 ± 5.3	28.1 ± 5.5	0.0116
Apo B† (g/L)	1.7 ± 0.5	0.80 ± 0.18	3.54 × 10^-56^
LDL-C (mmol/L)	6.8 ± 1.6	2.06 ± 0.62	2.7 × 10^-141^
Non–HDL-C (mmol/L)	7.4 ± 1.6	2.53 ± 0.67	5.92 × 10^-144^
Lp(a) (mg/dL)	28.3 ± 30.9	27.8 ± 31.0	0.851
Median Lp(a) (mg/dL)	13.0 (30.2)	10.7 (29.0)	0.562

Values are presented as mean ± SD or median (interquartile range [IQR]). *P* values comparing hypercholesterolemia and control subjects were obtained using 2-tailed unpaired Student's *t*-tests to compare sample means or Mann-Whitney U tests to compare median values.

Apo B, apolipoprotein B; BMI, body mass index; Lp(a), Lipoprotein (a); LDL-C, low-density lipoprotein cholesterol; non–HDL-C, non–high-density lipoprotein cholesterol.

∗ n = 252 (FH); 184 (control).

† n = 238 (FH); 257 (control).

### Levels of Lp(a) in patients with hypercholesterolemia vs control subjects

A Mann-Whitney U test was performed to compare Lp(a) levels in the patients with hypercholesterolemia vs controls. Median levels are reported in Table 1; no difference was identified (*P* = 0.617).

### Correlation between LDL-C and Lp(a)

**Figure 1:**
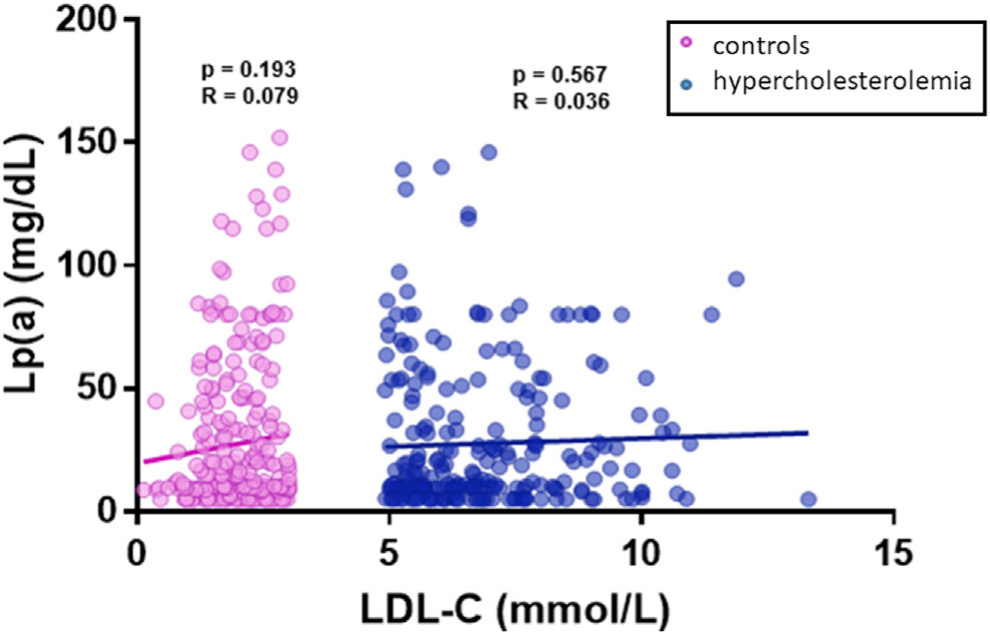
Correlation between plasma levels of LDL-C and Lp(a) in controls and patients with hypercholesterolemia. The relationship between LDL-C and Lp(a) in control (**pink**, n = 272) and hypercholesterolemia (**blue**, n = 256) cohorts was examined. A linear trendline, associated Pearson correlation coefficient and *P* value for the control (*r* = 0.079; *P* = 0.193) and FH cohorts (*r* = 0.036; *P* = 0.567) are displayed here. The observed trend indicates that there is no correlation between the plasma levels of the 2 lipoproteins in either cohort. FH, familial hypercholesterolemia; LDL-C, low-density lipoprotein cholesterol; Lp(a), Lipoprotein(a).

No correlation between Lp(a) and LDL-C was identified in either cohort (Fig. 1): for patients with hypercholesterolemia overall, *r* = 0.036 (*P* = 0.567) and for controls *r* = 0.079 (*P* = 0.193). When subdivided by genotype, no correlation was found between LDL-C and Lp(a) in the FH variant-positive group (N = 154; *r* = 0.093; *P* = 0.249), FH variant-negative group (N = 101; *r* = –0.020; *P* = 0.846), high PRS group (N = 64; *r* = –0.112; *P* = 0.378), or low PRS group (N = 187; *r* = 0.091; *P* = 0.213) (Supplemental Fig. S1).

### Correlation between non–HDL-C and Lp(a)

**Figure 2:**
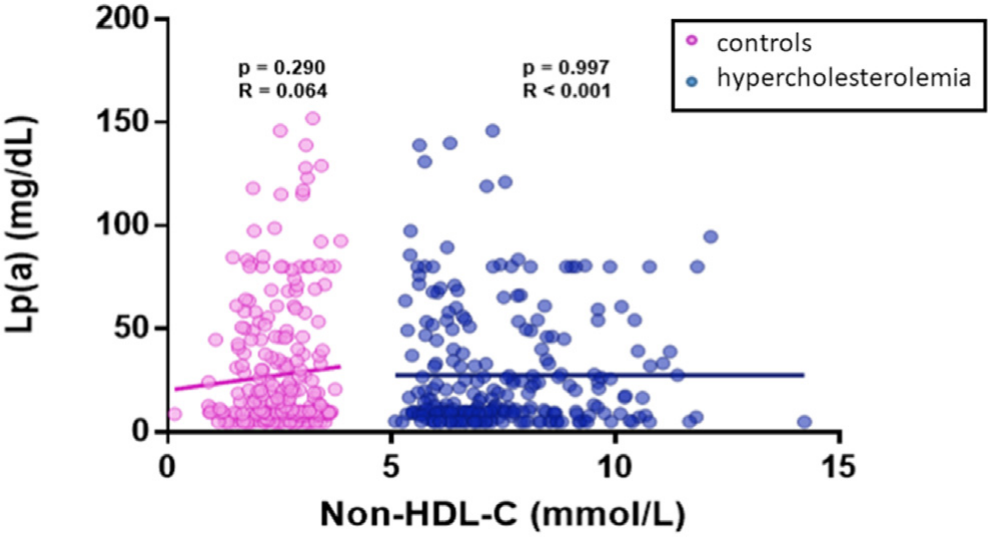
Correlation between non–HDL-C and Lp(a) in control and hypercholesterolemia patients. The relationship between non–HDL-C and Lp(a) in control (**pink**, n = 272) and hypercholesterolemia (**blue**, n = 256) cohorts was examined. A linear trendline, associated Pearson correlation coefficient and *P* value for the control (*r* = 0.064; *P* = 0.290) and FH cohorts (*r* < 0.001; *P* = 0.997) are displayed here. The observed trend indicates that there is no correlation between the plasma levels of the 2 variables in either cohort. Lp(a), Lipoprotein(a); non–HDL-C, non–high-density lipoprotein cholesterol.

No correlation between non-HDL-C and Lp(a) was identified in either controls (*r* = 0.064, *P* = 0.290) or patients (*r* < 0.001; *P* = 0.997) (Fig. 2). When subdivided by genotype, no correlations were found between non–HDL-C and Lp(a) in the FH variant-positive group (N = 154; *r* = 0.070; *P* = 0.390), FH variant-negative group (N = 101; *r* = –0.105; *P* = 0.297), high PRS group (N = 64; *r* = –0.169; *P* = 0.182), or low PRS group (N = 187; *r* = 0.066; *P* = 0.371), (Supplemental Fig. S2).

### Correlation between apo B and Lp(a)

**Figure 3:**
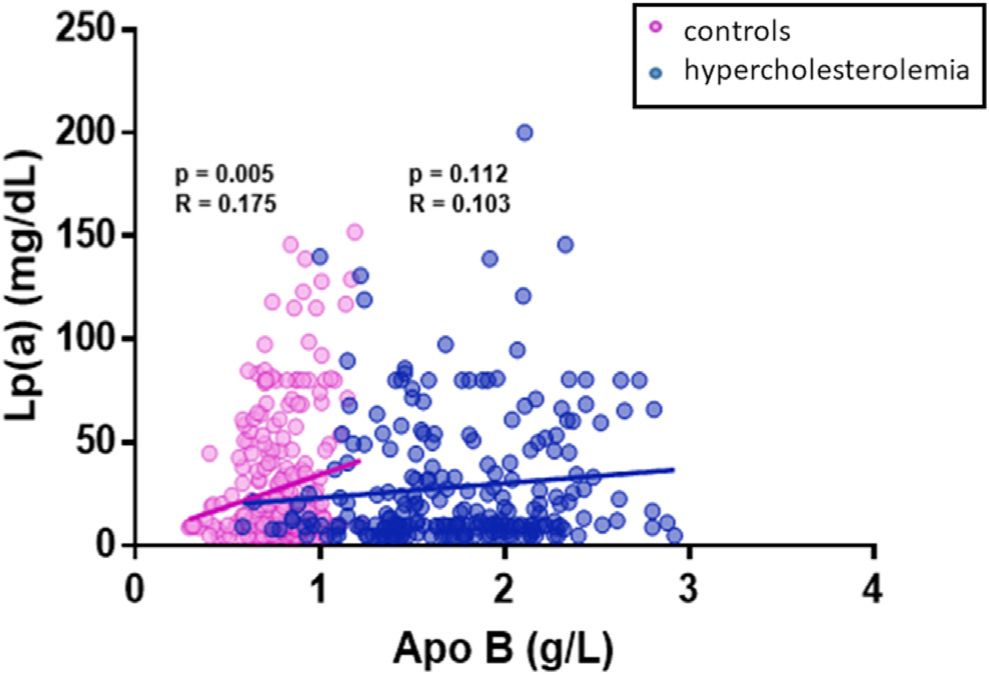
Correlation between apo B and Lp(a) in control and patients with hypercholesterolemia. The relationship between apo B and Lp(a) in control (**pink**, n = 257) and hypercholesterolemia (**blue**, n = 238) cohorts was examined. A linear trendline and the associated Pearson correlation coefficient and *P* value for the control (*r* = 0.175; *P* = 0.005) and FH cohorts (*r* = 0.103; *P* = 0.112) are displayed here. The observed trend indicates that apo B and Lp(a) are marginally correlated in the control group only, with no significant relationship observed in the FH cohort. apo B, apolipoprotein B; FH, familial hypercholesterolemia; Lp(a), Lipoprotein(a).

Apo B was only marginally correlated with Lp(a) in controls (*r* = 0.175; *P* = 0.005), and nonsignificantly in the patients (*r* = 0.103; *P* = 0.112) (Fig. 3). When subdivided by genotype, no correlation was found between apo B and Lp(a) in the FH-variant positive (N = 142; *r* = 0.092; *P* = 0.274), FH variant-negative (N = 96; *r* = 0.192; *P* = 0.061), or high PRS groups (N = 61; *r* = –0.163; *P* = 0.210). Apo B and Lp(a) showed weak borderline correlation in the low PRS group (N = 173; *r* = 0.185; *P* = 0.015) (Supplemental Fig. S3).

## Discussion

The principal findings of this observational study in patients with hypercholesterolemia are that Lp(a) levels are not elevated compared with controls; Lp(a) is not correlated with either LDL-C or non–HDL-C; and Lp(a) is only marginally correlated with apo B in control individuals. In patients with hypercholesterolemia, no relationship was observed between Lp(a) and LDL-C (*r* = 0.036; *P* = 0.567), non-HDL-C (*r* < 0.001; *P* = 0.997), or apo B (*r* = 0.103; *P* = 0.112). Findings were similar whether patients had pathogenic FH variants or polygenic hypercholesterolemia. Elevated Lp(a) does not appear to be a cause of FH in our clinic. In control individuals, no relationship was observed between Lp(a) and LDL-C (*r* = 0.079; *P* = 0.193) or non–HDL-C (*r* = 0.064; *P* = 0.290), although a weak correlation was observed between Lp(a) and apo B (*r* = 0.175; *P* = 0.005).

Superficially, clinicians and scientists might conjecture that Lp(a) levels would be elevated in HeFH and correlated with LDL-C and non–HDL-C levels, given the structural and biochemical overlaps between these entities; for example, they are all apo B containing. However, absence of correlation between Lp(a) and LDL-C is not surprising, as it is well established that Lp(a) is metabolically distinct from the other lipoprotein classes, with independent synthesis and catabolism pathways.^5^ Furthermore, Lp(a) particle numbers (in nmol/L), even among individuals with extremely high levels, are several orders of magnitude lower than LDL particle numbers (in mmol/L) and indeed those of all other apo B-containing and non–HDL lipoprotein species,^9^ which might explain the marginal-to-absent correlations with these other traits.

Previous research has found that the ratio of Lp(a)/apo B particles increased with the number of circulating Lp(a) particles.^9^ Because levels of apo B-containing lipoproteins are lower in control subjects than in those with hypercholesterolemia,^10^ Lp(a) may comprise a relatively higher proportion of these species, perhaps explaining the observed borderline weak correlation signal in control subjects but not in those with hypercholesterolemia. In general, LDL constitutes the vast majority of particles (> 80%) that contribute to non–HDL-C and apo B levels in patients both with and without hypercholesterolemia, whereas very low-density lipoproteins, remnant particles, and Lp(a) constitute only a minor proportion.^10^ It should thus not be surprising that these variables are essentially not correlated. Also, reported estimates that elevated Lp(a) levels may explain up to 25% of patients with apparent FH seem inflated,^11^ especially in light of our observations that the distribution of Lp(a) levels in patients with FH covers a wide range, including mostly low levels. Furthermore, the distribution of Lp(a) levels appears indistinguishable from that in control subjects (see especially Figs. 1 and 2), indicating that “elevated” Lp(a) levels are just as likely to be seen in patients with FH as in normolipidemic control individuals without FH. If high Lp(a) truly a “cause” of 25% of FH cases, mean Lp(a) levels would be higher in FH than controls, which is not the case in our cohort.

For decades, investigators who evaluated levels of Lp(a) in patients with HeFH reached varying conclusions. For instance, Utermann and colleagues reported in 1989 that Lp(a) levels were higher in HeFH patients than in control subjects but—more importantly—identified a synergistic interaction between these 2 variables on risk of ASCVD.^12^ The same investigators reported that Lp(a) levels were even higher in ultra-rare individuals with HeFH.^13^ In contrast, a 1991 study in large Utah families with HeFH reported no difference in Lp(a) levels among family members with and without FH-linked DNA variants.^14^ Examples of such disparities have persisted over subsequent decades. In 2014, Alonso and colleagues observed in the Spanish Familial Hypercholesterolemia Cohort (SAFEHEART) registry that Lp(a) levels were slightly, but significantly higher in 1960 patients with HeFH than 957 controls by 10%: that is, 23 vs 21 mg/dL.^15^ Whether this difference is clinically or biologically relevant is debatable, but the authors’ main finding was that patients with HeFH and ASCVD endpoints had significantly higher Lp(a) plasma levels than unaffected relatives and that Lp(a) was an independent predictor of cardiovascular disease.^15^

A concern with studying HeFH cohorts is that higher ASCVD incidence may result in ascertainment bias, which could skew the sampling toward those with elevated Lp(a). To mitigate against such potential bias, Trinder and colleagues studied Lp(a) levels in conjunction with disease-causing FH variants in an unbiased population sample: namely, the UK Biobank.^6^ These authors found no difference in Lp(a) levels among 391 individuals with and 37,486 individuals without FH-causing variants, suggesting that HeFH from genetically impaired LDLR function does not cause elevated Lp(a),^6^ which is consistent with our findings. However, elevated Lp(a) among those with HeFH consistently raised risk of ASCVD endpoints in virtually every study.^16^, ^17^, ^18^, ^19^ Thus, elevated Lp(a) probably increases the likelihood that an individual with HeFH will be clinically recognized because they and their relatives are at higher risk of ASCVD.

Our subgroup analyses indicate that the general absence of correlation between Lp(a) and LDL-C, non–HDL-C, and apo B is independent of genotype, whether hypercholesterolemia is monogenic caused by pathogenic variant or has a polygenic basis. It is believed that hypercholesterolemic patients with a high PRS have many variants that subtly affect both synthesis and catabolism of LDL.^2^, ^3^, ^4^ Absence of correlation between Lp(a) and either LDL-C or non–HDL-C indicates that, in these patients, Lp(a) is produced or catabolized independently of other lipoproteins, extending the observations from those with monogenic HeFH caused by pathogenic variants affecting LDLR-mediated catabolism of LDL particles. Overall, these genetic results indicate that Lp(a) has distinct metabolic determinants compared with LDL and the broader spectrum of apo B-containing lipoproteins comprising the non-HDL family of particles.

### Limitations

Our study has some limitations. First, Lp(a) levels were not controlled according to apo(a) isoform size, which requires a highly specialized, relatively inaccessible assay.^5^ Because larger apo(a) isoforms are more readily catabolized and are associated with lower Lp(a) levels, some clinical biochemists propose that controlling for apo(a) isoform size would provide a more accurate picture of Lp(a) levels and their clinical implications. Second, the clinical assay used to quantify Lp(a) is relatively less precise at extreme levels. Third, the Friedewald formula can occasionally attribute to the calculated LDL-C value cholesterol that is carried by Lp(a), especially when Lp(a) levels are very high. However, if this were a major issue, we would have noted a correlation between Lp(a) and LDL-C levels, which we did not. Fourth, our sample—although well characterized—was relatively small, although a post hoc power analysis showed we have power of 0.84 to detect a clinically relevant 10 mg/dL (or 30%) Lp(a) increase in Lp(a) in patients with FH compared with controls (α = 0.05), although this power is reduced to 0.20 to detect a borderline relevant 3 mg/dL (or 10%) increase in Lp(a). Fifth, apo B levels in cases and controls seem to overlap to a greater degree than LDL-C and non–HDL-C, possibly reflecting the need for a validated imputation algorithm specifically for apo B and emphasizing a potential limitation of using the correction factor for LDL-C. Sixth, we did not assess the association of elevated Lp(a) levels with incident ASCVD outcomes in our samples, which was beyond the scope of our hypothesis testing for biochemical correlations only. Finally, because our study was performed in a single centre, it would be ideal to repeat and expand the evaluation with a prospective, multicentre, and multiethnic group study.

## Conclusions

Our observations are consistent with the idea that Lp(a) and LDL-C levels are independent of each other in patients with hypercholesterolemia, specifically in patients with FH. Elevated Lp(a) was not a cause of FH in our cohort. Patients with HeFH did not have significantly elevated Lp(a) levels compared with controls. Marked elevations in LDL-C—along with elevated apo B and non–HDL-C—are thus not predictive of Lp(a) levels. Our findings are consistent with the current consensus that Lp(a) amplifies the increased risk of ASCVD that is already present among individuals with genetic hypercholesterolemia and that these individuals must have their Lp(a) determined as part of comprehensive risk assessment. As there are no specific therapies at present for elevated Lp(a), the general treatment principle would be to improve global risk maximally by focusing on modifiable risk factors such as LDL-C, blood pressure, glycemia, smoking, and weight. For patients with HeFH for whom therapy with PCSK9 inhibitors is contemplated, the moderate Lp(a) lowering ability of these agents^20^^,^^21^ might be a consideration in those with concomitant Lp(a) elevations. Novel therapies that dramatically lower Lp(a) specifically^22^^,^^23^ might eventually prove to be beneficial for hypercholesterolemic patients who concurrently have elevated Lp(a) levels.
