# Supplementary material for: Lipoprotein(a) in Familial Hypercholesterolemia
Source: CJC Open. 2023 Sep 30;6(1):40–6. doi: 10.1016/j.cjco.2023.09.018 (PMC10837708; doi:10.1016/j.cjco.2023.09.018)
Supplement: Supplemental Table and Figures [file mmc1.docx]

**Supplementary files**

**Lipoprotein (a) in familial hypercholesterolemia**

Erin O. Jacob, BMSc^a,b^, Adam D. McIntyre, BSc^b^, Jian Wang, MD^b^, and Robert A. Hegele, MD*^a,b,c^

^a^ Department of Biochemistry, Schulich School of Medicine and Dentistry, Western University; London, ON, Canada, N6A 5K8

^b^ Robarts Research Institute, Schulich School of Medicine and Dentistry, Western University; London, ON, Canada, N6A 5K8

^c^ Department of Medicine, Schulich School of Medicine and Dentistry, Western University; London, ON, Canada, N6A 5K8

**Supplementary Table S1.** Clinical and demographic characteristics of control and hypercholesterolemia subjects subdivided by gender. Values are presented as mean ± SD or median (IQR). P-values comparing hypercholesterolemia and control cohorts were obtained using two-tailed unpaired t-tests to compare sample means, or Mann-Whitney U tests to compare medians.

|  | **Male patients**  **(N = 118, 46.1%)** | **Female patients**  **(N = 138, 53.9%)** | **P-value**  **(male vs female)** | **Male controls**  **(N = 166, 61.0%)** | **Female controls (N = 106, 39.0%)** | **P-value (male vs female)** | **P-value (male patient vs control)** | **P-value (female patient vs control)** |
| --- | --- | --- | --- | --- | --- | --- | --- | --- |
| **Age (years)** | 41.0 ± 14.1 | 46.1 ± 14.7 | 0.005 | 50.7 ± 14.2 | 51.2 ± 15.9 | 0.829 | 3.01E-08 | 0.0120 |
| ***BMI (kg/m^2^)** | 27.3 ± 5.2 | 26.3 ± 5.4 | 0.138 | 28.4 ± 4.8 | 27.8 ± 6.5 | 0.504 | 0.117 | 0.113 |
| **˚Apo B (g/L)** | 1.8 ± 0.5 | 1.7 ± 0.5 | 0.916 | 0.797 ± 0.182 | 0.804 ± 0.175 | 0.956 | 1.57E-22 | 1.52E-35 |
| **LDL-C (mmol/L)** | 6.8 ± 1.7 | 6.8 ± 1.5 | 0.915 | 2.03 ± 0.63 | 2.10 ± 0.61 | 0.915 | 9.02E-61 | 1.97E-82 |
| **Non-HDL-C (mmol/L)** | 7.4 ± 1.7 | 7.4 ± 1.5 | 0.875 | 2.52 ± 0.70 | 2.55 ± 0.64 | 0.875 | 4.64E-63 | 2.40E-82 |
| **Lp(a) (mg/dL)** | 25.3 ± 26.3 | 30.8 ± 34.3 | 0.148 | 27.2 ± 29.9 | 28.7 ± 32.7 | 0.148 | 0.575 | 0.624 |
| **Median Lp(a) (mg/dL)** | 10.4 (24.0) | 15.3 (34.3) | 0.23 | 10.7 (29.1) | 10.5 (28.3) | 0.23 | 0.897 | 0.441 |

*Control: n = 112 (M), n = 72 (F); FH: n = 117 (M), n = 135 (F)

**˚**Control: n = 158 (M), n = 99 (F); FH: n = 109 (M), n = 129 (F)

Abbreviations: Apo B, apolipoprotein B; BMI, body mass index; IQR, interquartile region; LDL-C, low-density lipoprotein cholesterol; Lp(a), lipoprotein (a); non-HDL-C, non-high-density lipoprotein cholesterol; SD, standard deviation.

**Supplementary Figure S1. Correlations between plasma levels of LDL-C and Lp(a) in hypercholesterolemia patients divided by genotype.** The relationship between plasma LDL-C and Lp(a) was identified and divided by genotype: (A) monogenic FH variant-positive (N = 154 , r = 0.093, P = 0.249); (B) monogenic FH variant-negative (N = 101, r = -0.020, P = 0.846), (C) high PRS (≥1.96) (N = 64, r = -0.112, P = 0.376); (D) low PRS (<1.96) (N = 187, r = 0.091, P = 0.213). The observed trend indicates no correlation between the plasma levels of the two lipoproteins regardless of genotype.

Abbreviations: FH, familial hypercholesterolemia; LDL-C, low-density lipoprotein cholesterol; Lp(a), Lipoprotein (a); PRS, polygenic risk score.

**Supplementary Figure S2. Correlations between plasma levels of non-HDL-C and Lp(a) in patients hypercholesterolemia divided by genotype.** The relationship between plasma non-HDL-C and Lp(a) was identified and divided by FH genotype: (A) monogenic FH-variant positive (N = 154, r = 0.070, P = 0.390); (B) monogenic FH-variant negative (N = 101, r = -0.105, P = 0.297), (C) high PRS (≥1.96) (N = 64, r = -0.169, P = 0.182); (D) low PRS (<1.96) (N = 187, r = 0.066, P = 0.371). The observed trend indicates no correlation between the plasma levels of the two variables regardless of genotype.

Abbreviations: FH, familial hypercholesterolemia; non-HDL-C, non-high-density lipoprotein cholesterol; Lp(a), Lipoprotein (a); PRS, polygenic risk score.

**Supplementary Figure S3. Correlations between plasma levels of apo B and Lp(a) in patients with hypercholesterolemia divided by genotype.** The relationship between plasma apo B and Lp(a) was identified and divided by FH genotype: (A) monogenic FH variant-positive (N = 142, r = 0.092, P = 0.274); (B) monogenic FH variant-negative (N = 96, r = 0.192, P = 0.061), (C) high PRS (≥1.96) (N = 61, r = -0.163, P = 0.210); (D) low PRS (<1.96) (N = 173, r = 0.185, P = 0.015). The observed trend indicates a potential positive correlation between apo B and Lp(a), with a statistically significant result present in the low PRS group only.

Abbreviations: FH, familial hypercholesterolemia; apo, apolipoprotein; Lp(a), Lipoprotein (a); PRS, polygenic risk score.
